# Putting the brakes on mammary tumorigenesis: Loss of STAT1 predisposes to intraepithelial neoplasias

**DOI:** 10.18632/oncotarget.371

**Published:** 2011-12-19

**Authors:** Christine Schneckenleithner, Zsuzsanna Bago-Horvath, Helmut Dolznig, Nina Neugebauer, Karoline Kollmann, Thomas Kolbe, Thomas Decker, Dontscho Kerjaschki, Kay-Uwe Wagner, Mathias Müller, Dagmar Stoiber, Veronika Sexl

**Affiliations:** ^1^ Institute of Pharmacology and Toxicology, University of Veterinary Medicine, Vienna, Austria; ^2^ Clinical Institute of Pathology, Medical University of Vienna, Austria; ^3^ Institute of Medical Genetics, Medical University of Vienna, Austria; ^4^ Biomodels Austria, University of Veterinary Medicine, Vienna and Biotechnology in Animal Production, IFA-Tulln, University of Natural Resources and Applied Life Sciences, Vienna; ^5^ Max F. Perutz Laboratories, Department of Genetics, Microbiology and Immunobiology, University of Vienna, Austria; ^6^ Eppley Institute for Research in Cancer and Allied Diseases, University of Nebraska Medical Center, Omaha, Nebraska; ^7^ Institute of Animal Breeding and Genetics and Biomodels Austria, University of Veterinary Medicine, Vienna, Austria; ^8^ Ludwig Boltzmann Institute for Cancer Research, Vienna, Austria; ^9^ Institute of Pharmacology, Medical University of Vienna, Austria

**Keywords:** Stat1, MIN, IRF1, mammary cancer, tumorsurveillance

## Abstract

Multiparous *Stat1^−/−^* mice spontaneously develop mammary tumors with increased incidence: at an average age of 12 months, 55% of the animals suffer from mammary cancer, although the histopathology is heterogeneous. We consistently observed mosaic expression or down-regulation of STAT1 protein in wild-type mammary cancer evolving in the control group. Transplantation experiments show that tumorigenesis in *Stat1^−/−^* mice is partially influenced by impaired CTL mediated tumor surveillance. Additionally, STAT1 exerts an intrinsic tumor suppressing role by controlling and blocking proliferation of the mammary epithelium. Loss of STAT1 in epithelial cells enhances cell growth in both transformed and primary cells. The increased proliferative capacity leads to the loss of structured acini formation in 3D-cultures. Analogous effects were observed when *Irf1^−/−^* epithelial cells were used. Accordingly, the rate of mammary intraepithelial neoplasias (MINs) is increased in *Stat1^−/−^* animals: MINs represent the first step towards mammary tumors. The experiments characterize STAT1/IRF1 as a key growth inhibitory and tumor suppressive signaling pathway that prevents mammary cancer formation by maintaining growth control. Furthermore, they define the loss of STAT1 as a predisposing event via enhanced MIN formation.

## INTRODUCTION

The signal transducers and activators of transcription (STATs) are an intensely studied family of transcription factors that have been recognized as critical mediators of cytokine and growth factor receptor signaling, required for cell proliferation, survival and differentiation [1, 2]. Activation of STATs is frequently observed in different cancer entities and it has been postulated that deregulation of these factors may be involved in tumorigenesis. STAT1 is constitutively expressed throughout the entire development of the mammary gland. Its phosphorylation pattern – elevated in virgin glands, low throughout gestation and lactation, rising again at late involution – is unique, although the exact function of phosphorylation is unclear [3]. STAT1 is expressed in the epithelial compartment of the mammary gland, suggesting it has an active role in epithelial cells [4]. STAT1 is of particular interest in mammary cancer as it is known to possess an independent prognostic significance in human breast cancer: high activation of STAT1 has been reported to correlate with an overall longer and relapse-free survival [5, 6]. Furthermore, treatment of human mammary tumor cells with cytostatic drugs has been shown to induce STAT1 activation, resulting in enhanced apoptosis [7, 8]. Recent work in mouse models of neu/ERBB2-induced breast cancer has underlined STAT1's tumor suppressive role [9, 10]. By crossing *Stat1^fl/fl^* mice with *MMTV-neu-IRES-cre* mice, Klover *et. al.* showed that tumor onset is significantly accelerated in Stat1*^fl/fl^ × MMTV-neu-IRES-cre* mice compared to STAT1-expressing wild-type controls. This conclusion suggests that STAT1 has an autonomous role in neu/ERBB2-induced mammary tumor formation. The second report did not discriminate between the intrinsic and the immunological contribution of STAT1-deficiency but came nevertheless to the conclusion that *Stat1^−/−^ × ERBB2/neu* mice develop mammary tumors significantly faster than control mice. Together, the studies unequivocally defined STAT1 as a tumor suppressor in mammary cancer.

STAT1's tumor suppressing properties may be related to cell-intrinsic effects as STAT1 has been shown to block proliferation and to be involved in the induction of apoptosis [11-15]. Furthermore, *Stat1^−/−^* mice have a severely compromised immune system due to their lack of IFN-signaling [16, 17] as well as to an impaired cytotoxic NK-cell and CTL capacity [18, 19]. The contributions of these different components to mammary tumor surveillance are to date poorly understood. Moreover, all previous studies have been based on oncogene-driven mammary tumor formation in the absence of STAT1.

We now report for the first time that loss of STAT1 alone is sufficient to cause pregnancy-associated mammary cancer in BALB/c mice, independent of any other transgenic oncogene. By transplanting *Stat1^−/−^* mammary glands into wild type recipient mice and vice versa we reveal that STAT1 contributes to the formation of mammary tumors through cell-intrinsic as well as immunological activities. *Stat1^−/−^* mammary epithelial cells exhibit enhanced proliferation, which might facilitate the development of mammary intraepithelial neoplasias (MINs) and subsequently also invasive mammary tumors. We suggest that STAT1/IRF1 acts in a linear axis to block growth. It is known that cytotoxic T-cells are impaired in *Stat1^−/−^* mice and we also characterize cytotoxic T-cells as major mediators of mammary tumor surveillance.

## RESULTS

### STAT1 deficiency is sufficient to cause mammary cancer

**Figure 1:**
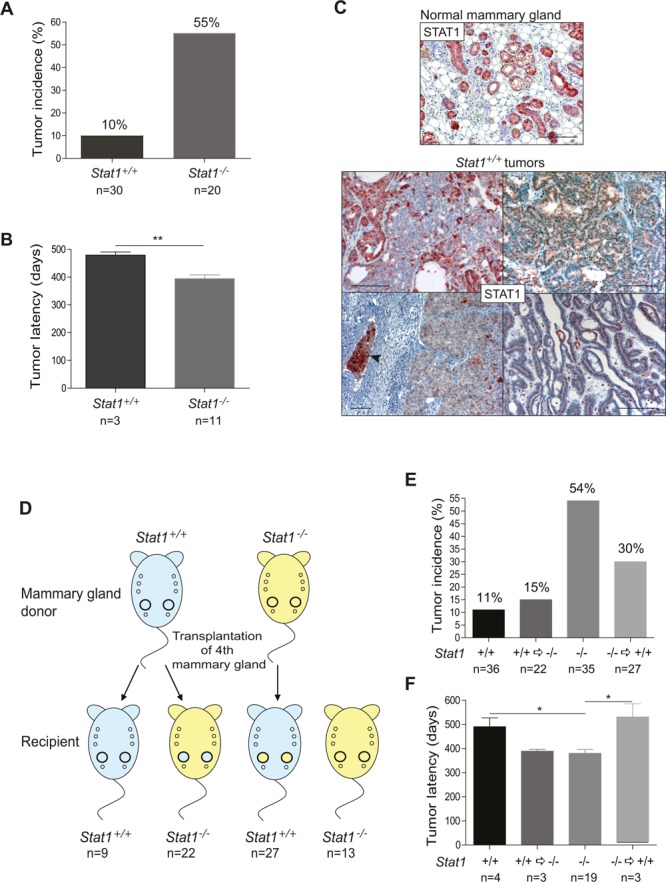
(A-C) Loss of STAT1 increases incidence and decreases latency of mammary tumors (A) Incidence and (B) latency of spontaneously occurring, parity-induced mammary tumors in *Stat1*^+/+^ (n=30) and *Stat1*^−/−^ (n=20) mice. Tumor development was monitored for up to 20 months. (C) STAT1-stained histological sections of a normal mammary gland from a multiparous wild type mouse after involution (upper panel) and of *Stat1*^+/+^ mammary tumors (bottom panel). *Stat1*^+/+^ tumors display mosaic expression and partial downregulation of STAT1 protein. The arrow indicates a vascular invasion. Scale bars: 100 μm. **(D-F) Absence of STAT1 in the immune system and also in the mammary gland tissue influences mammary tumor formation**. (D) Experimental setup of mammary gland transplantation experiment. Mammary gland tissue of *Stat1*^−/−^ mice was transplanted into the cleared fat pad of the 4^th^ mammary gland of *Stat1*^+/+^ mice (n=27), or *Stat1*^+/+^ mammary glands were transplanted into *Stat1*^−/−^ animals (n=22). Control groups: *Stat1*^+/+^ tissue transplanted into *Stat1*^+/+^ animals (n=9), non-transplanted *Stat1*^−/−^ animals (n=13). Mice were bred constantly and checked for mammary tumor growth for up to 20 months. On average, each animal had 7 litters. (E) Incidence and (F) latency of spontaneously occurring, parity-induced mammary tumors in all mice in the transplantation experiment. Tumors were classified into four groups: *Stat1*^+/+^ tumors in *Stat1*^+/+^ mice, *Stat1*^+/+^ tumors in *Stat1*^−/−^ mice, *Stat1*^−/−^ tumors in *Stat1*^−/−^ mice and *Stat1*^−/−^ tumors in *Stat1*^+/+^ mice.

BALB/c mice are predisposed to develop mammary tumors and are therefore suitable to study spontaneous mammary tumorigenesis [11]. To evaluate the role of STAT1 in the spontaneous development of mammary tumors we crossed *Stat1^−/−^* mice into the BALB/c genetic background. Groups of wild-type BALB/c and *Stat1^−/−^*/c female mice were kept under breeding conditions and regularly controlled by palpation for mammary tumor formation. Within an average of one year, 55% of the multiparous *Stat1^−/−^* mice had developed mammary tumors. In the control group, only 10% had mammary tumors and disease onset was significantly later (*Stat1^−/−^*: 394.5 days ± 13.52 and *Stat1^+/+^*: 479.3 days ± 11.46; ^**^P = 0.0089; values represent mean ± SEM) (Fig. 1A and 1B). No mammary tumors were detected in wild-type or *Stat1^−/−^* nulliparous animals over a period of 20 months. Interestingly, all tumors that evolved in wild-type mice showed a mosaic expression and partial down-regulation of STAT1 protein (Fig. 1C). Loss or down-regulation of STAT1 was restricted to tumor cells and was not observed in the normal mammary tissue surrounding the cancer.

### Mammary epithelial transplantation uncovers a mammary gland cell-intrinsic and an immunological contribution of STAT1 to the development of mammary tumors

To test whether these results stemmed from an intrinsic action of STAT1 within the mammary epithelium rather than simply being related to tumor-promoting changes triggered by the impaired immune system of the *Stat1^−/−^* animals, we transplanted *Stat1^−/−^* mammary epithelial tissue into the cleared fat pad of the 4^th^ mammary gland of *Stat1^+/+^* mice, and vice versa (see scheme in Fig. 1D). To control for tumor onset provoked by the transplantation procedure itself, we additionally transplanted *Stat1^+/+^* mammary tissue into *Stat1^+/+^* mice. Moreover, a group of *Stat1^−/−^* mice was maintained under identical conditions in a non-transplanted setting. All animals were bred constantly to accelerate mammary tumor development. Animals were sacrificed when any evolving mammary tumor reached a diameter of one centimeter or earlier if there were signs of disease such as weight loss, scrubby fur or reduced mobility. All transplanted mammary glands – irrespective of whether a visible tumor had evolved – were subjected to histological analysis to assess the success of the transplantation procedure and to study spontaneous tumorigenesis.

Immunohistochemical staining for STAT1 revealed a successful outgrowth in 36% of *Stat1^−/−^* donor tissue in *Stat1^+/+^* recipient mice upon transplantation, whereas in the *Stat1^+/+^* => *Stat1^+/+^* setting the take rate reached 90% (Fig. S1A and S1B). FACS analysis did not show any significant differences in mammary stem cell populations between wild type and *Stat1^−/−^* animals, eliminating the possibility that this cell compartment contributed to the repopulation frequency (Fig. S2A). The analysis of freshly transplanted mammary glands uncovered a dense infiltration with CD3^+^ T-lymphoid cells and NKp46^+^ NK-cells, irrespective of the genotype (Fig. S2B). Although we used syngenic mice in the experiment – all animals were back-crossed to BALB/c – an inflammatory infiltrate is unavoidable due to tissue damage during the transplantation procedure. We assumed that *Stat1^−/−^* mice accepted the transplanted tissue better due to their partially impaired immune system.

Spontaneous tumor development was monitored over a period of 20 months in our cohort of transplanted animals. STAT1 expression was surveyed by immunohistochemical staining and PCR-analysis. Non-transplanted mammary glands where analysed for tumor development and served as an internal control. The observed tumor incidence and latency are summarized in Figs 1E and 1F. Spontaneous tumors developed within 20 months from non-transplanted tissue in 19/35 (54%) of the *Stat1^−/−^* mice and in 4/36 (11%) of the *Stat1^+/+^* mice. During this time, 3/22 (14%) of the *Stat1^−/−^* animals displayed tumors that originated from the transplanted *Stat1^+/+^* mammary glands. Even when adjusted for the success rate of transplantation (90%), the tumor incidence did not exceed 15% in this group. In the reverse experiment, 3/27 *Stat1^−/−^* tumors evolved in *Stat1^+/+^* mice, translating into a 30% tumor incidence after adjustment for the significantly lower transplantation success rate.

In summary, our observations enable us to conclude that STAT1 suppresses tumor formation in the mammary epithelial cells themselves. Tumor incidence increases upon transplantation of *Stat1^−/−^* mammary glands into *Stat1^+/+^* recipients compared to a *Stat1^+/+^* => *Stat1^+/+^* scenario (30% versus 11%). We also deduce that the STAT1-deficient immune system contributes to and accelerates carcinogenesis. *Stat1^+/+^* as well as *Stat1^−/−^* mammary tumors occurred with an increased incidence in a *Stat1^−/−^* environment compared to in *Stat1^+/+^* surroundings (15% versus 11% for *Stat1^+/+^* tumors; 54% versus 30% for *Stat1^−/−^* tumors). Consistently, tumor development occurred with a significantly shorter latency in a *Stat1^−/−^* environment, irrespective of whether spontaneous tumor development or carcinomas evolving after transplantation are compared (Fig. 1F).

### Loss of STAT1 favors mammary intraepithelial neoplasias (MINs)

**Figure 2:**
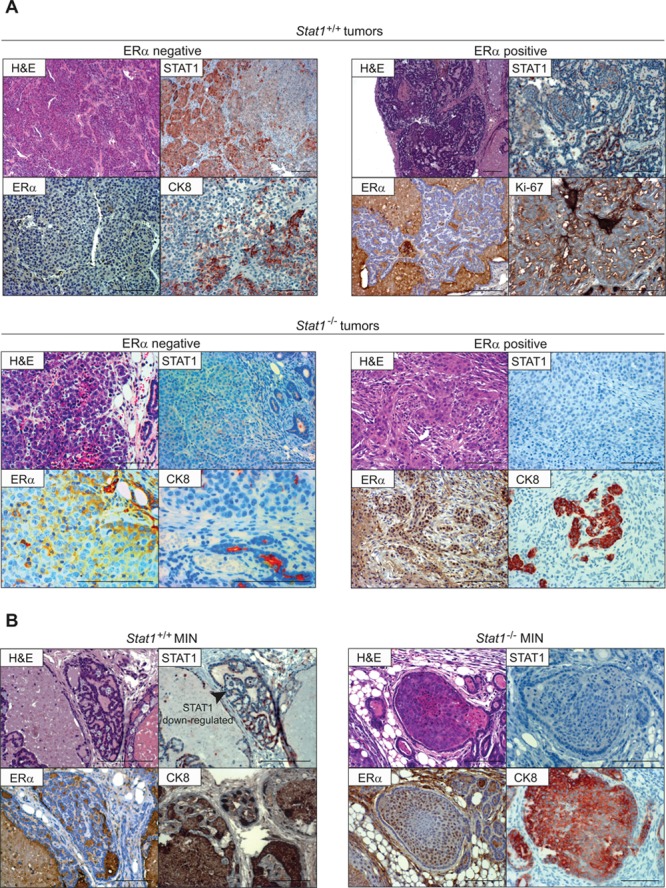
(A) Both, Stat1+/+ and Stat1−/− mammary tumors are heterogenous Representative pictures of immunohistochemical characterization of two *Stat1*^+/+^ (top panel) and two *Stat1*^−/−^ mammary tumors (bottom panel). Scale bars: 100 μm. **(B) Loss of STAT1 correlates with MIN formation**. Representative pictures of immunohistochemical characterization of one low-grade *Stat1*^+/+^ (left panel) and one high-grade *Stat1*^−/−^ MIN (right panel). *Stat1*^+/+^ MINs display down-regulation of STAT1 protein, as indicated by the arrow. Scale bars: 100 μm; ERα: estrogen receptor alpha; CK8: cytokeratin 8.

An overview of the histopathological and immunohistochemical features of the tumors that arose in our animal cohort is provided in Table I. Fig. 2A depicts representative panels of the immunohistochemical characterization of two *Stat1^+/+^* and two *Stat1^−/−^* tumors. The tumors were characterized either as neoplasisas *in situ* (mammary intraepithelial neoplasia, MIN) or as invasive ductal carcinomas. No lobular carcinomas were diagnosed. Grading was performed according to Elston and Ellis [20]. Invasive features were present in 62% of all *Stat1^−/−^* and in 80% of all *Stat1^+/+^* tumors. We did not detect any genotype-related pattern with regard to grading of tubule formation, nuclear polymorphism or mitotic count. However, we found a high incidence of MIN (8/13) in *Stat1^−/−^* tumors: 4 of low grade and 4 of high grade (Table I). In contrast, 2/5 *Stat1^+/+^* tumors displayed only low-grade MINs and no high-grade MIN could be detected. Importantly, the two cases of MIN detected in *Stat1^+/+^* mice had largely lost STAT1 protein expression (Fig. 2B). These observations revealed an increased incidence of MIN upon loss of STAT1.

**Table I:** Histopathological and immunohistochemical analysis and classification of mammary tumors Histological sections of all available mammary tumors were analyzed and mouse mammary tumors characterized according to a standard nomenclature used to classify human breast carcinomas. Invasive carcinomas were graded according to Elson and Ellis [20]. 1: well-differentiated breast cells, cells generally appear normal and do not grow rapidly, cancer arranged in small tubules; 2: moderately-differentiated breast cells, have characteristics between Grade 1 and Grade 3 tumors; 3: poorly differentiated breast cells, cells do not appear normal and tend to grow and spread more aggressively. Tubule formation (% of carcinoma composed of tubular structures) − 1: > 75%; 2: 10-75%; 3: less than 10%. Nuclear pleomorphism − 1: small, uniform cells; 2: moderate increase in size and variation; 3: marked variation. Mitosis count − 1: up to 7; 2: 8 to 14; 3: 15 or more. Mammary intraepithelial neoplasia (MIN) − -: not detected; low grade; moderate grade; high grade. Estrogen receptor alpha (ERα) – Reiner score [36], i.e. 0-2: negative; 3: low positive; 4-5: moderate positive; 6-7: strong positive. Human epidermal growth factor receptor 2 (HER2) – 0-1: negative; 2: low positive; 3: strong positive.

Number	Genotype Tumor	Genotype Mouse	Histological Classification	Invasiveness				MIN	ERa	HER2
				Tubule formation	Nuclear pleomorphism	Mitosis count	Grading			
**1**	*Stat1^−/−^*	*Stat1^−/−^*	ductal/with medullary features	3	3	3	3	-	0	0
**2**	*Stat1^−/−^*	*Stat1^−/−^*	ductal	2	2	1	1	-	0	0
**3**	*Stat1^−/−^*	*Stat1^−/−^*	ductal	3	3	3	3	-	0	0
**4**	*Stat1^−/−^*	*Stat1^−/−^*	ductal	2	3	3	3	high grade	4(60%/2)	1
**5**	*Stat1^−/−^*	*Stat1^−/−^*	ductal	3	3	3	3	high grade	3(30%/1)	0
**6**	*Stat1^−/−^*	*Stat1^−/−^*	ductal/with metaplastic features	3	3	2	3	high grade	3(20%/1)	0
**7**	*Stat1^−/−^*	*Stat1^−/−^*	intraductal with microinvasion					low grade	0	1~2
**8**	*Stat1^−/−^*	*Stat1^−/−^*	intraductal-papillary					low grade	3(10%/1)	0
**9**	*Stat1^−/−^*	*Stat1^−/−^*	intraductal with microinvasion					low grade	3(50%/1)	0
**10**	*Stat1^−/−^*	*Stat1^−/−^*	Intraductal-papillary					low grade	0	0
**11**	*Stat1^−/−^*	*Stat1^+/+^*	ductal	2	2	1	1	-	0	1
**12**	*Stat1^−/−^*	*Stat1^+/+^*	ductal	3	3	2	3	-	4(60%/2)	0
**13**	*Stat1^−/−^*	*Stat1^+/+^*	intraductal					high grade	4(60%/2)	1
**14**	*Stat1^+/+^*	*Stat1^−/−^*	ductal/with metaplastic features	3	3	2	3	-	0(<10%)	0
**15**	*Stat1^+/+^*	*Stat1^+/+^*	ductal	1	1	1	1	-	5(50%/3)	2
**16**	*Stat1^+/+^*	*Stat1^+/+^*	ductal	2	2	3	2	-	0	1
**17**	*Stat1^+/+^*	*Stat1^+/+^*	ductal	1	1	1	1	low grade	0(<10%)	0
**18**	*Stat1^+/+^*	*Stat1^+/+^*	intraductal with microinvasion					low grade	0	n.a.

### The STAT1/IRF1 axis is implicated as having an important role in MIN formation, regulating the proliferation of mammary epithelial cells

**Figure 3:**
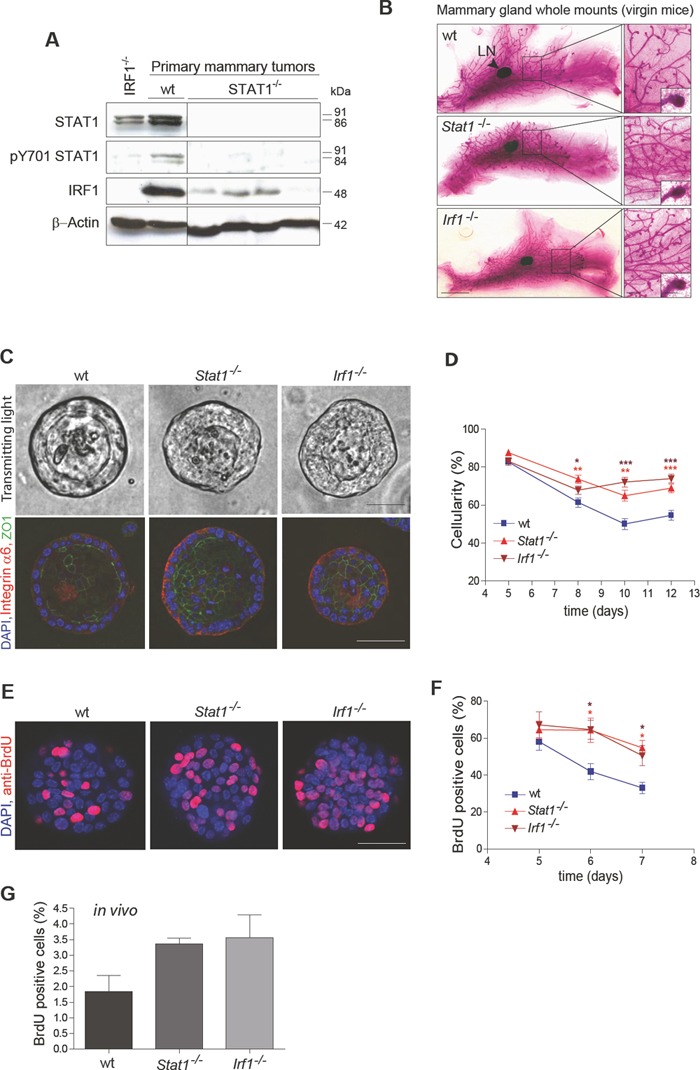
(A, B) Loss of STAT1 might cause mammary tumor formation by down-regulating IRF1 (A) Western blotting of mammary tumor samples. Low expression of IRF1 protein correlates with STAT1 expression. (B) Representative pictures of whole mounts of mammary glands from 50 day-old virgin wild-type (wt), *Stat1*^−/−^ and *Irf1*^−/−^ mice. *Stat1*^−/−^ and *Irf1*^−/−^ glands show an increased density of ductal structures but no differences in end duct formation. Scale bars: 0.5 cm, 500 μm, 100 μm. LN: lymph node. **(C-F) Mammary epithelial cells of *Stat1*^−/−^ and *Irf1*^−/−^ mice show enhanced proliferation**. (C+D) In a 3D culture assay, primary mammary epithelial cells derived from *Stat1*^−/−^ and *Irf1*^−/−^ mice formed spheres with increased cellularity compared to wild-type controls. (C) Representative bright-field microscopy pictures (top panel) and fluorescence microscopy pictures (bottom panel) from 8 day-old spheres of different genotypes. (Blue) DAPI; (red) Integrin α6; (green) ZO1. Scale bars: 50 μm. (D) Cellularity of spheres was measured at different time points. For details of calculations see SI Materials and Methods. n ≥ 33 per genotype. (E+F) To measure proliferation, spheres were exposed to BrdU for 24h, before fixation. (E) Representative fluorescence microscopy pictures of 6 day-old spheres derived from wt, *Stat1*^−/−^ and *Irf1*^−/−^ epithelial cells. (Blue) DAPI; (red) anti-BrdU. Scale bars: 50 μm. (F) Percentage of BrdU-positive cells was calculated at different times of growth. n ≥ 18; 1 n = 1 sphere. (D+F) Statistical analysis: asterisks represent significances to the wild type. No significant differences could be detected between any *Stat1*^−/−^/*Irf1*^−/−^ pair. Data are representative of three independent experiments. (G) To measure proliferation *in vivo*, BrdU was injected intraperitoneally into mice. Mammary gland cells were isolated and percentages of BrdU-positive mammary gland cells after 3 days of BrdU administration were measured using the BD FACS-Canto II FACS device with the BD FACS Diva software (Beckton Dickinson). n = 3 per genotype.

Loss of the STAT1 downstream transcription factor IRF1 has been reported in MIN cases of human breast cancer [21]. Moreover, the loss of heterozygosity at the IRF1 gene locus has been found to be a frequent event in human breast cancer [22]. It thus seems possible that STAT1 and IRF1 act in a common axis to suppress MIN and subsequently mammary tumor formation [23, 24]. Initial evidence for this hypothesis was provided by the analysis of STAT1 and IRF1 protein expression in primary mouse mammary tumor tissue: the levels of IRF1 protein correlated with the expression levels of STAT1. Furthermore, IRF1 expression in *Stat1^−/−^* tumors was shown to be low (Fig. 3A). To evaluate whether both *Stat1^−/−^* and *Irf1^−/−^* mice are predisposed to develop mammary tumors, whole mounts of mammary glands were analyzed. It was noteworthy that already at the virgin state *Stat1^−/−^* and *Irf1^−/−^* mice displayed an increased amount of ductal structure compared to wild-type controls (Fig. 3B and S3), although MIN could not be detected at that age in these *Stat1^−/−^* and *Irf1^−/−^* mice.

To investigate the role of STAT1 and IRF1 in MIN formation, we employed an *in vitro* three-dimensional culture system, which allowed us to monitor the formation of polarized acini from single mammary epithelial cells [25]. Mammary epithelial cells were prepared from glands of 6- to 8- week-old virgin wild-type, *Stat1^−/−^* and *Irf1^−/−^* mice and analyzed in this 3D culture system. Acini developed irrespective of the genotype and with no significant difference in total volumes of the spheres (Fig. S4). Remarkably, *Stat1^−/−^* and *Irf1^−/−^* acini displayed a significantly higher cellularity at all time points compared to wild-type controls (Figs 3C, top panel and 3D). Whereas mammospheres of wild-type controls were formed by a single monolayer, immunofluorescent staining showed a less organized cell array and partial epithelial bi-layering in *Stat1^−/−^* and *Irf1^−/−^* spheres (Fig. 3C, bottom panel and Videos S1A, B and C). The increased cellularity could not be attributed to decreased apoptosis as there were no detectable signs of cleaved caspase-3 activity, even at early time points (day 2) when the lumen of the acini starts to evolve (Fig. S5). This finding is in line with the observations made by Jechlinger [25], who described that lumina form without epithelial cells undergoing apoptosis. The polarity of the *Stat1^−/−^* and *Irf1^−/−^* acini was unaltered as staining for the basal marker Integrin α6 did not reveal any major changes. Furthermore, we failed to detect any changes in the formation of tight junctions by staining for Zona Occludens-1 (ZO1), a protein that binds directly to occludins and is a bona fide marker for tight junctions (Fig. 3C, bottom panel). However, examining BrdU incorporation revealed a significantly increased proliferation rate in *Stat1^−/−^* and *Irf1^−/−^* spheres compared to wild type controls *in vitro* (Fig. 3E and 3F). This was confirmed by analysis of mammary epithelial proliferation *in vivo*. For this purpose, BrdU was injected intraperitoneally into mice for a period of 3 days. Isolated mammary glands were digested and cells in single-cell suspensions were stained with anti-BrdU antibody. Quantification of BrdU-positive cells by flow cytometry confirmed the enhanced proliferation of *Stat1^−/−^* and *Irf1^−/−^* mammary epithelial cells (Fig. 3G).

### Mammary tumor formation is under the control of cytotoxic T-lymphocytes (CTLs)

**Figure 4:**
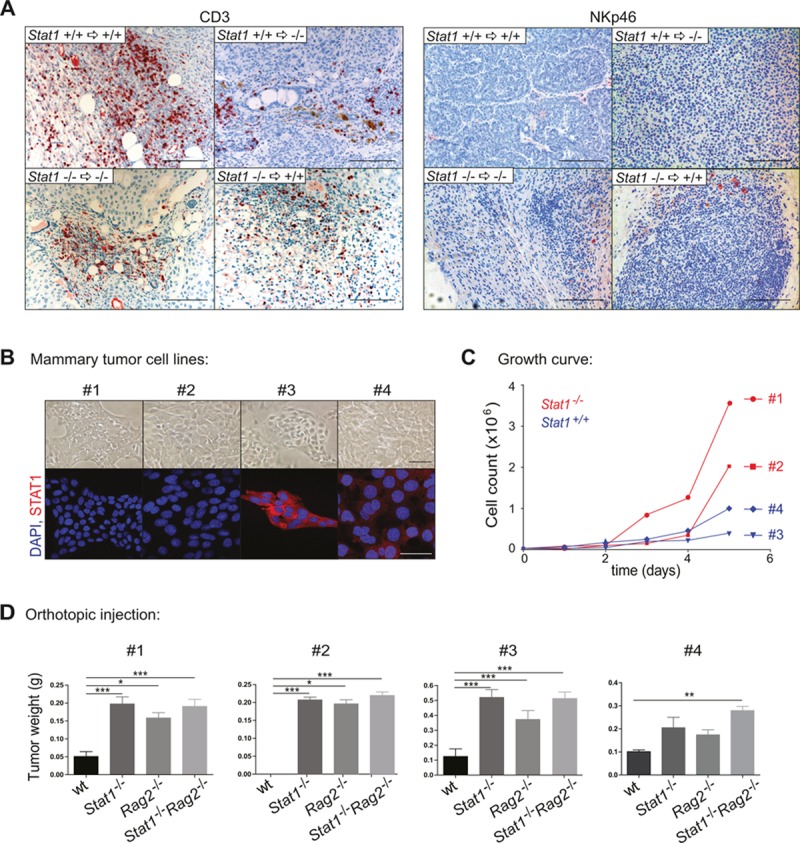
Mammary tumor growth is mainly controlled by cytotoxic T-cells and only to a minor extent by NK-cells (A) Histological sections of spontaneously occurring mammary tumors of the transplant experiment were stained with CD3 and NKp46 antibodies. Tumors are listed in four groups: *Stat1*^+/+^ tumors in *Stat1*^+/+^ mice, *Stat1*^+/+^ tumors in *Stat1*^−/−^ mice, *Stat1*^−/−^ tumors in *Stat1*^−/−^ mice and *Stat1*^−/−^ tumors in *Stat1*^+/+^ mice. Scale bars: 100 μm. (B) Bright-field (top panel) and fluorescence (bottom panel) microscopy pictures of tumor cell lines derived from two *Stat1*^−/−^ (#1, #2) and two *Stat1*^+/+^ (#3, #4) spontaneous mammary tumors. (Blue) DAPI; (red) STAT1. Scale bars: 50 μm. (C) Growth curve of mammary tumor cell lines. (D) Mammary tumor cell lines were orthotopically injected into wt, *Stat1*^−/−^, *Rag2*^−/−^ and *Stat1*^−/−^*Rag2*^−/−^ animals. Tumor weights of different groups were compared. n ≥ 5 per genotype. All data are representative of two independent experiments.

Our transplantation studies revealed that the absence of STAT1 from the immune system significantly enhances tumor incidence and shortens the latency of mammary tumor formation. STAT1 is believed to have an essential role in CTL- and NK-cell cytotoxicity; both lymphoid lineages are important mediators of tumor surveillance. Immunohistochemical staining for CD3 and NKp46 verified a dense infiltration of all mammary tumors with cytotoxic T-cells, whereas in all tumors NK-cells were rare and not consistently detectable (Fig. 4A). To clarify the contribution of NK and/or cytotoxic T cells to tumor surveillance, we generated mammary tumor cell lines. Four cell lines (#1, #2: *Stat1^−/−^*; #3, #4: *Stat1^+/+^*) were established during the course of our study, all of which displayed an epithelial-like phenotype (Fig. 4B). Of note, cell lines lacking STAT1 had a proliferative advantage *in vitro* and *in vivo* over *Stat1^+/+^* lines (Fig. 4C and S6A-C). 5×10^5^ cells were orthotopically injected into the mammary glands of wild type, *Stat1^−/−^*, *Rag2^−/−^* or *Stat1^−/−^Rag2^−/−^* mice. As *Rag2^−/−^* animals lack T cells and rely on NK cells for tumor surveillance, this experiment allowed us to determine the individual contribution of T and NK cells to tumor surveillance. The experiment was terminated and tumor weights determined when the first tumors reached approximately 1 cm in diameter. The *Stat1^−/−^* cell line #2 failed to induce tumors in wild-type mice and tumor formation was restricted to immuno-compromised animals. This observation is consistent with previous findings that tumors evolving in immunodeficient hosts (such as *Stat1^−/−^*) are more immunogenic and not immuno-edited and may be rejected in immuno-competent surroundings [26].

Fig. 4D summarizes the data from orthotopic tumor injections. We found a consistently and significantly enhanced tumor growth in *Stat1^−/−^* mice compared to wild-type controls, irrespective of the cell line injected. Compared to *Rag2^−/−^* animals, tumor growth in *Stat1^−/−^* or *Stat1^−/−^Rag2^−/−^* hosts was not significantly accelerated, indicating that the NK-cell compartment played at most a minor part in restricting proliferation of the transplanted tumor cells. Support for this conclusion came from a comparison of wild-type recipients with *Rag2^−/−^* mice. Although *Rag2^−/−^* mice contain functionally competent NK-cells, tumor growth in these mice was significantly increased, indicating that the NK cell compartment was not capable of significantly limiting tumor expansion. In summary, the data indicate a dominant role for cytotoxic T cells in tumor surveillance in spontaneously occurring, parity-induced mammary tumors.

## DISCUSSION

We show that the transcription factor STAT1 has a tumor-suppressing function against the formation of parity-induced, spontaneous mammary tumors. We show that STAT1 deficiency significantly increases tumor incidence in BALB/c mice as well as decreasing disease latency. STAT1 acts in a dual manner. It sustains proper CTL activity and thus ensures tumor surveillance, while also exerting growth inhibitory and tumor suppressing effects via its downstream regulator IRF1.

Our findings are in line with recent reports on the role of STAT1 in the context of ERBB2/neu/HER2 induced mammary cancer development [9, 10]. Using different mouse models, both groups concluded that STAT1 suppresses ERBB2/neu/HER2 tumor formation. In our cohort of mice we characterized tumors that evolved spontaneously in the absence of a driving oncogene. The fact that only 2/13 of the mammary cancers expressed ERBB2/neu/HER2 indicates that STAT1's tumor-suppressing effect is not limited to ERBB2/neu/HER2-induced tumorigenesis. The effect of STAT1 loss is also not restricted to tumors that display a particular pattern of expression of the estrogen receptor (ER): 7/13 of the spontaneously evolving *Stat1^−/−^* mammary cancers were ER^+^, while the remaining cases did not express detectable levels of the ER protein. These findings show conclusively that STAT1 is a global tumor suppressor that acts independently of a distinct oncogenic driver.

The importance of STAT1 as a tumor suppressor was underlined by the finding that all *Stat1^+/+^* mammary cancers had partially lost or down-regulated STAT1 protein expression. Similar observations were reported in human patients, where low levels of STAT1 activation have been linked to a poor prognosis [5, 27]. The selection pressure to down-regulate or lose STAT1 in tumor cells may have more than one cause as the loss of STAT1 has several important consequences: the tumor cells lose responsiveness towards interferon-mediated growth inhibition (interferons are important players in tumor surveillance). Moreover, STAT1 is the key regulator of MHC class I expression. By losing STAT1 the cells may down-regulate MHCI and thus escape CTL-mediated tumor surveillance [28, 29]. As NK cells play a negligible part in tumor surveillance, MHCI loss represents a clear-cut advantage. CTLs dominated the immune infiltrate in the tumor sections and their importance was further verified in transplant studies. In the absence of the adaptive immune system, tumor development occurred rapidly and was significantly accelerated, whereas the presence of NK cells did not interfere with tumor onset. Hence, mammary tumors are partially able to escape immune control by down-regulating STAT1. In *Stat1^−/−^* animals, the selective pressure to escape immune control is less important as the mice have several defects that limit their capability to exert effective tumor surveillance. As a consequence, mammary cancer formation is increased in a *Stat1^−/−^* micro-environment, irrespective of whether the epithelial cells themselves express STAT1. The finding underlines the role of the immune system in spontaneously evolving mammary cancer. Although a pro-inflammatory environment and T-cell infiltration may exert a tumor promoting effect, our orthotopic injection experiments unequivocally show that there is a tumor suppressing function that opposes the tumor promoting effect of the immune infiltrate.

Loss or down-regulation of STAT1 confers an additional advantage, i.e. accelerated cell proliferation. Under normal conditions, STAT1 puts the brakes on cell proliferation in mammary epithelial cells, presumably via the transcription factor IRF1. Two lines of evidence support this assumption. First, whole mounts of *Stat1^−/−^* as well as of *Irf1^−/−^* mammary glands display increased amounts of ductal structures compared to wild type controls, even in the virgin state. Secondly, BrdU incorporation studies *in vitro* and *in vivo* confirm an increased DNA synthesis and thus enhanced growth in mammary epithelial cells of both *Stat1^−/−^* and *Irf1^−/−^* mice.

As a consequence of enhanced cell proliferation, MIN formation was frequently found in *Stat1^−/−^* mice. This finding was also reflected in 3D-culture experiments, in which the cell composition of *Stat1^−/−^* and *Irf1^−/−^* acini was less organized. The original report describing the 3D system used the two oncogenes *MYC* and *Kras^G12D^* to characterize the occurrence of highly proliferative depolarized spheres resembling MIN. As expected, the alterations observed in *Stat1^−/−^*- and *Irf1^−/−^*-derived spheres are less pronounced than those occurring in the presence of *MYC* and *Kras^G12D^*. In the absence of STAT1 and IRF1, polarity of the spheres is preserved and a lumen – albeit smaller – is maintained in most cases. Therefore, additional alterations are required to allow mammary tumor formation. These additional alterations are most probably triggered by hormonal stimulation and changes occurring during lactation in the breast tissue, as we failed to detect spontaneous tumorigenesis in nulliparous mice. Our conclusion that STAT1/IRF1 act in a linear axis to block growth is in perfect accordance with reports by others that attribute a negative regulatory role of IRF1 on cell growth: enforced expression of IRF1 in mammalian cell lines slows or even halts proliferation [30-32].

Cancer formation is a multi-level process during which a cell successively acquires several genetic or epigenetic alterations that ultimately cooperate to allow the development of a malignant tumor. One of the alterations required to initiate the process is loss of growth control. Our findings support a model in which STAT1 represents a critical safeguard that preserves growth control in mammary epithelial cells. The absence of STAT1 facilitates cell proliferation and therefore MIN formation, which represents the first step on the road from normal breast tissue towards invasive breast cancer [33].

The exact characterization of the early alterations has a significant potential for use in preventative therapy of invasive breast cancer and might lead to the development of novel immune-modulatory strategies to combat the disease. The critical effect of STAT1 is not restricted to any distinct tumor type but is of global relevance.

## MATERIAL AND METHODS

### Mice

All animals were maintained in spf quality at the University of Veterinary Medicine, Vienna. C.Cg-Stat1^tm1^ (*Stat1^−/−^*) [16], C.Cg-Irf1^tm1Mak^ (*Irf1^−/−^*) [34] and C.129S6-Rag2^tm1Fwa^ (*Rag2^−/−^*) [35] mice have been described previously. C.Cg-Stat1^tm1^-Rag2^tm1Fwa^ (*Stat1^−/−^Rag2^−/−^*) mice were crossed at the University of Veterinary Medicine, Vienna. Animal experiments were discussed and approved by the institutional ethics committee and undertaken in conformance with Austrian laws.

### Orthotopic injection of mammary tumor cell lines

Mammary tumor cell lines #1-#4 were derived from spontaneous mammary tumors. For orthotopic injection of mammary tumor cell lines, mice were anaesthetized and depilated on the belly. 5×10^5^ cells were injected via the nipple into the fat pad of the 4^th^ and 5^th^ mammary gland of wild type, *Stat1^−/−^*, *Rag2^−/−^* and *Stat1^−/−^Rag2^−/−^* mice. The mice were sacrificed when the tumors reached one centimeter in diameter.

### Histology and immunohistochemistry

The following antibodies were used for immunostaining in accordance with to the manufacturers' protocol: STAT1 (Santa Cruz, #sc-592), CD3 (Neomarkers, RM9107), NKp46 (BioLegend, #137601), ERα (Santa Cruz, #sc-542), CK8 (Developmental Studies Hybridoma Bank, TROMA-I) and Ki67 (Novocastra, NCL-Ki67). Nuclear counterstaining was performed with hematoxylin. Pictures were taken on a Zeiss AxioImager Z1 microscope system with a CCD camera using the software PixelNK Capture 3.0.

### Immunofluorescence

For immunofluorescent staining, cells or 3D cultures were incubated with primary antibodies against STAT1 (Santa Cruz, #sc-592), integrin α6 (Millipore, #MA1378) or ZO1 (Zymed Laboratories, #33-9100), followed by incubation with Alexa546-conjugated or Alexa488-conjugated goat antibodies against rat or mouse IgG (Molecular Probes, #A11081 and #A11001). Incubation times were prolonged for 3D cultures to ensure complete staining. Cells were counterstained with DAPI and imaged using a confocal laser-scanning microscope (Carl Zeiss LSM 700, Occulare 10x, 40x Oil) using the software ZEN 2009 LE.

### Statistics

All statistical analysis was performed using GraphPad Prism 4. Differences were assessed for statistical significance by One-way ANOVA using the Tukey's Multiple Comparison Test. For Figure 1B only, the unpaired t-test was used. Error bars represent mean ± SD. *P* values are considered as follows: <0.05: *; <0.01: **; <0.001: ***.

## Supplementary Videos, Figures and Methods





Video S1. 3-dimensional reconstruction of mammospheres Images are representative for6 day-old (A) wt, (B) *Stat1*^−/−^ and (C) *Irf1*^−/−^ spheres. Pictures were taken on a confocal laser scanning microscope (Carl Zeiss LSM 700, Occulare 10x, 40x Oil). Intervals of 2 μm were used for image collection. (Red) DAPI; (green) Integrin α6.
