# Supplementary material for: Putting the brakes on mammary tumorigenesis: Loss of STAT1 predisposes to intraepithelial neoplasias
Source: Oncotarget. 2011 Dec 19;2(12):1043–54. doi: 10.18632/oncotarget.371 (PMC3282066; doi:10.18632/oncotarget.371)
Supplement: Supplementary file 4 [file oncotarget-02-1043-s004.pdf]

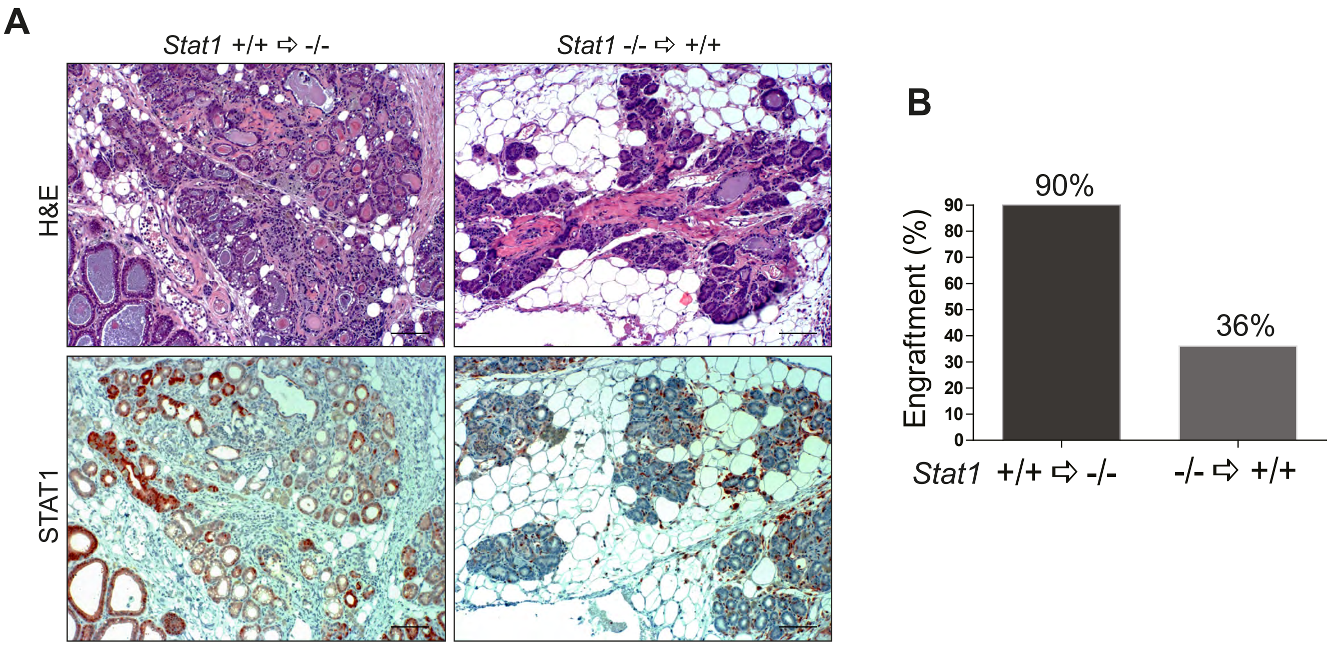

Fig. S1. Verification of successful engraftment of transplanted mammary gland tissue. (A) Histological sections of transplanted mammary glands were stained for H&E and STAT1 to verify successful engraftment of transplants. Left panel: *Stat1*$^{+/+}$ mammary gland tissue was transplanted into a *Stat1*$^{-/-}$ mouse; right panel: *Stat1*$^{-/-}$ mammary gland tissue was transplanted into a *Stat1*$^{+/+}$ mouse. Scale bars: 100 µm. (B) Rate of successful engraftment of transplanted mammary gland tissue in *Stat1*$^{+/+}$ and *Stat1*$^{-/-}$ animals. Transplant groups: *Stat1*$^{+/+}$ tissue in *Stat1*$^{-/-}$ mice, *Stat1*$^{-/-}$ tissue in *Stat1*$^{+/+}$ mice.

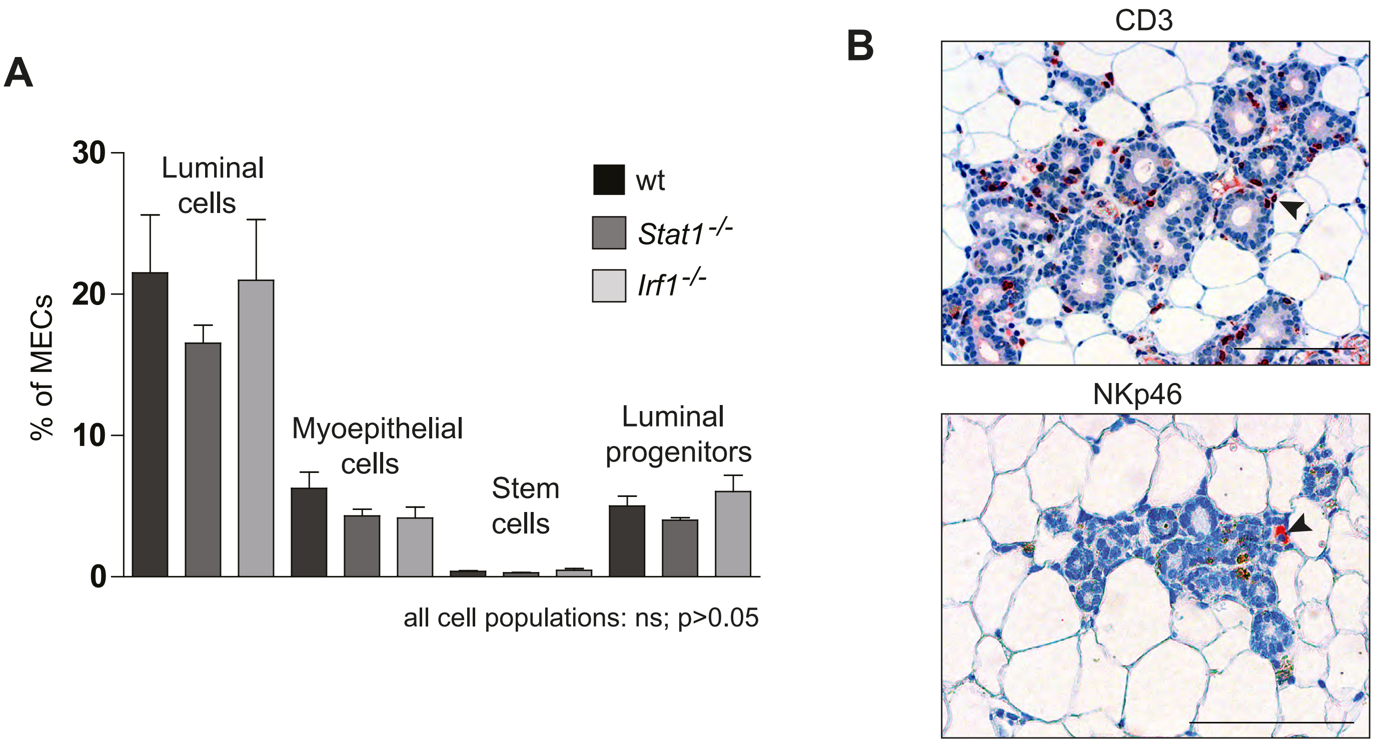

Fig. S2. Successful engraftment of transplanted mammary gland tissue is influenced by CTLs and NK-cells but not by differences in mammary stem cell populations. (A) Flow cytometric analysis of mammary cells from wt, *Stat1*$^{-/-}$ and *Irf1*$^{-/-}$ mice. Lumial cells: CD24^{hi}CD49f^{lo}, myoepithelial cells: CD24^{lo}CD49f^{hi}, stem cells: CD24^{mid}CD49f^{hi}, Luminal progenitors: CD24^{hi}CD49f^{lo}CD61^{+}. (B) CD3- and NKp46-stained histological sections of transplanted mammary glands show infiltration with CTLs and NK-cells. Scale bars: 100 µm.

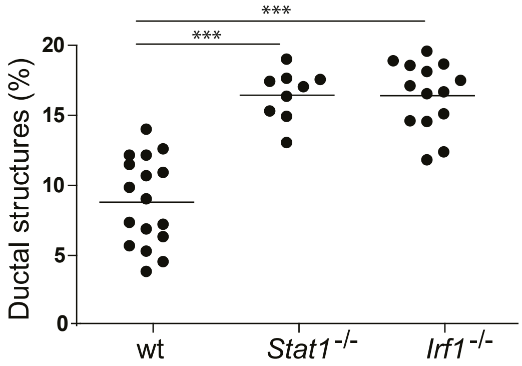

Fig. S3. Quantitative analysis of ductal density from mammary gland whole mounts. Pictures of 2.5x magnification were taken and analyzed using ImageJ 1.37a software. $n \geq 9$.

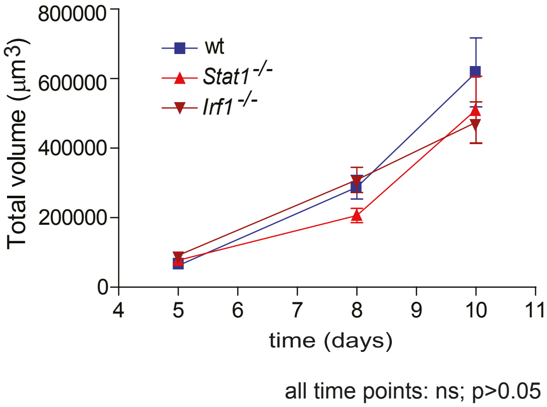

Fig. S4. Total volume of 3D-cultured spheres derived from wt, *Stat1*$^{-/-}$ and *Irf1*$^{-/-}$ mammary epithelial cells at different time points of growth. Total volume (V_T) was calculated by means of the formula $V_T = 4/3 * r_T^3$.

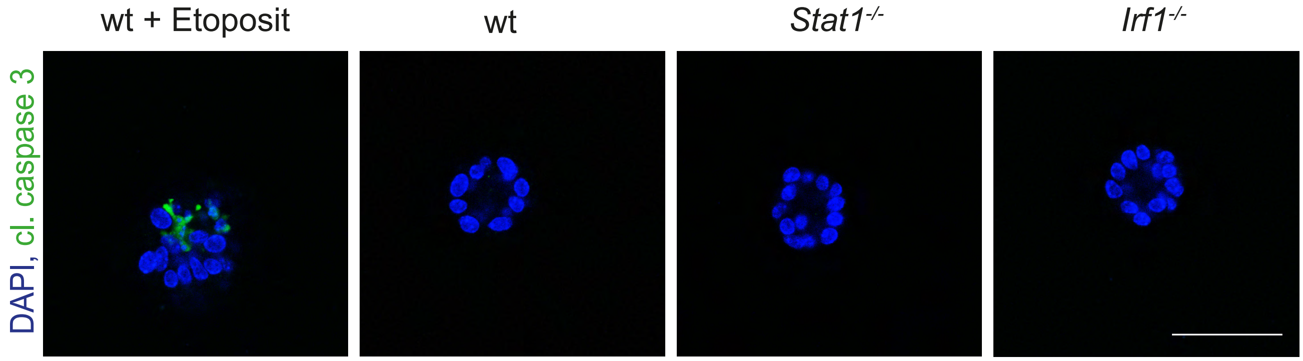

Fig. S5. Mammospheres for lumen without involvement of apoptotic mechanisms. Floureszence microscopy pictures from 2 day-old spheres of different genotypes. To ensure a positive cleaved caspase 3 staining, wildtype mammospheres were treated with 50 µm Etoposit for 24h. (Blue) DAPI; (green) Cleaved caspase 3. Scale bar: 50 µm.

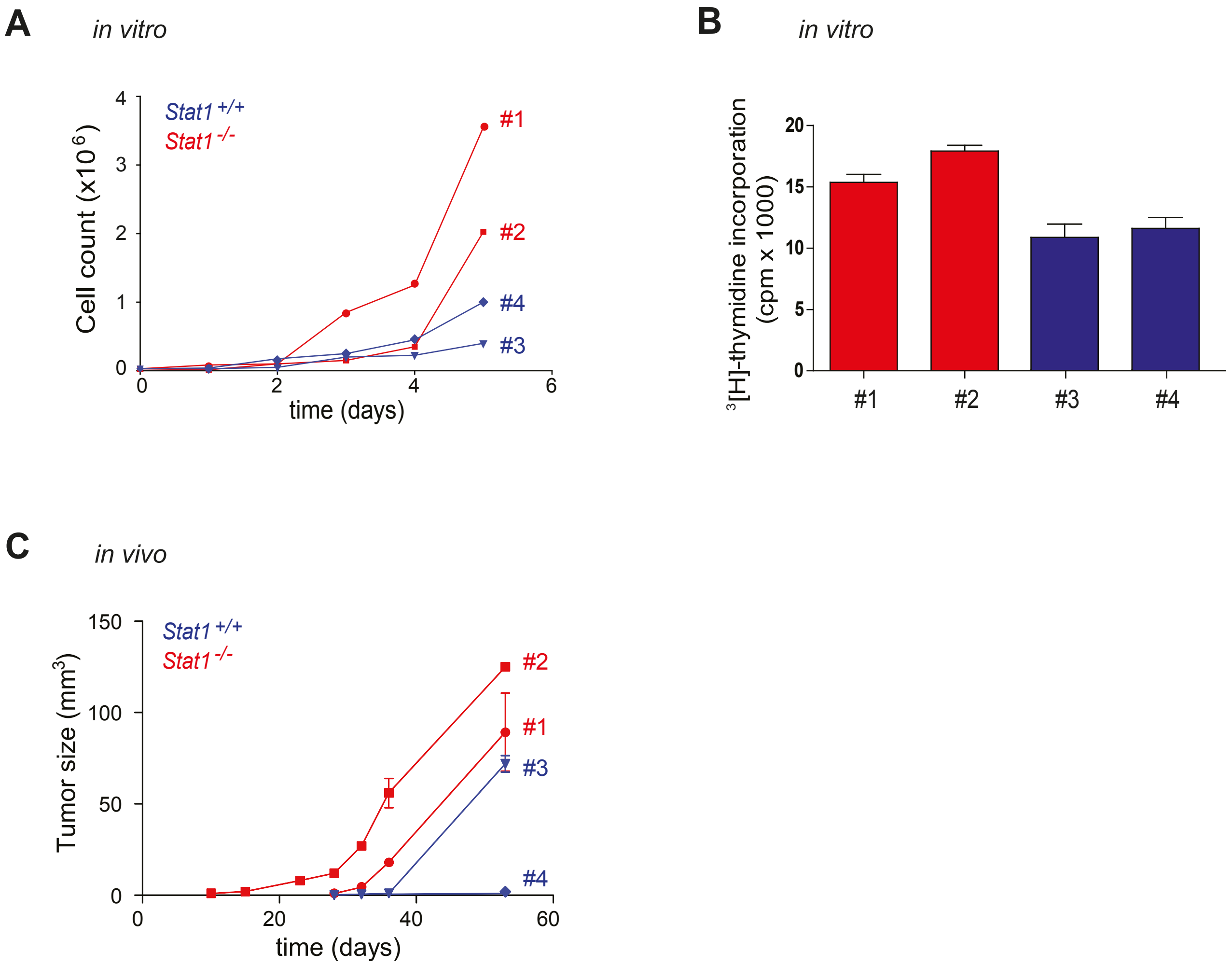

Fig. S6. ***Stat1**$^{-/-}$* **mammary tumor cell lines show increased proliferation *in vitro* and *in vivo*.** (A) Growth curve of mammary tumor cell lines #1-#4. (B) [H^3]-thymidine incorporation of mammary tumor cell lines #1-#4. (C) *In vivo* proliferation of mammary tumor cell lines #1-#4. 2x10^5 cells of each cell line were orthotopically injected into *Stat1*$^{-/-}$ mice. Tumor growth was measured at different time points with tumor size calculated by means of the formula V=3/4*π*L*W*H. n ≥ 4 per cell line.
