# Supplementary material for: Putting the brakes on mammary tumorigenesis: Loss of STAT1 predisposes to intraepithelial neoplasias
Source: Oncotarget. 2011 Dec 19;2(12):1043–54. doi: 10.18632/oncotarget.371 (PMC3282066; doi:10.18632/oncotarget.371)
Supplement: Supplementary file 5 [file oncotarget-02-1043-s005.pdf]

Supporting Information:

Materials and Methods

Mammary epithelial transplant procedure. Procedures for transplantation of tissues into cleared mammary fat pads have been described recently [1]. In brief, small pieces (1-2 mm^3) of mammary epithelium from the 4th mammary gland were taken from 3-month-old virgin females and implanted into the cleared fad pad of the 4th mammary gland of 3-week-old female recipient mice. Eight weeks after surgery, transplant recipients were housed with males for impregnation and monitored regularly for mammary tumor growth by palpation. A mouse was sacrificed when any evolving mammary tumor reached one centimeter in diameter or earlier if there were signs of disease.

3D-cultures of primary mouse mammary epithelial cells. 3D culture assays of primary mammary epithelial cells were performed as described previously [2] with minor alterations. The tissue from two mammary glands was placed in 5 ml digestion medium (DMEM with L-glutamine supplemented with 1 M HEPES-Buffer to a final concentration of 25 mM, 150 U/ml Collagenase type 3 (Worthington, #LS004182) and 20 µg/ml Liberase (Roche applied science, #05401119001)) and digested for 6 h at 37°C. Following 40 min of treatment with 0.25% Trypsin-EDTA, cells were seeded onto collagen-coated plates. One day after plating, cells were harvested and counted. 10.000 cells were resuspended in 50 µl of "Cultrex 3D Culture MatrixTM Basement Membrane Extract" (TREVIGEN, #3445-005-01) and carefully dispensed onto 4-well chamber slides (Millipore, #PEZGS0416). After solidification at 37°C, gels were supplemented with Mammary Epithelial Cell Medium (BulletKit, Lonza, #CC-3150) and maintained at 37°Cin a CO_2 incubator until analysis.

Calculating the cellularity of mammospheres: Spheres were assumed to be round. Circles were drawn around the entire sphere and at the borders of the lumen; total volume (V_T) and lumen volume (V_L) were calculated by means of the formula $V_{T/L}=4/3*r_{T/L}^3$, so cellularity was $C=V_T-V_L$. Pictures were taken on an Olympus iX5 using the cell^F software.

BrdU incorporation assay. The APC BrdU Flow Kit (BD Pharmingen^{TM}) was used to determine proliferation. For *in vitro* experiments, 3D cultures were incubated with 10 ng/ml BrdU for 24 hours and fixed and processed according to the manufacturer's instructions with minor changes. Incorporated BrdU was detected by APC-conjugated anti-BrdU antibody and cell nuclei were counterstained with DAPI. The stainings were visualized under a confocal laser scanning microscope (Carl Zeiss LSM 700, Occulare 10x, 40x Oil). Spheres in random microscopic fields were taken to determine the percentage of cells with nuclear BrdU incorporation from the total cell number within one sphere. For *in vivo* experiments, mice were injected intraperitoneally with 4 mg BrdU solution. After 3 days the mice were sacrificed and mammary glands were dissected and digested as described above. Cells were fixed and processed according to the manufacter's instructions. APC-conjugated anti-BrdU-stained cells were analyzed by flow cytometry using the BD FACS-Canto II FACS device and BD FACS Diva software (Beckton Dickinson). Epithelial cells not exposed to BrdU but stained with anti-BrdU antibody were used as appropriate negative control to avoid counting background signals as false positive.

1. Young LJT: The cleared mammary fad pad and the transplantation of mammary gland morphological structures and cells. In: *Methods in mammary gland biology and breast cancer research.* Edited by Ip MM, Asch BB. Heidelberg: Springer 2000: 67-74.
2. Jechlinger M, Podsypanina K, Varmus H. Regulation of transgenes in three-dimensional cultures of primary mouse mammary cells demonstrates oncogene dependence and identifies cells that survive deinduction. Genes Dev. 2009; 23:1677-1688.
